# Locating the Binding Sites of Pb(II) Ion with Human and Bovine Serum Albumins

**DOI:** 10.1371/journal.pone.0036723

**Published:** 2012-05-04

**Authors:** Ahmed Belatik, Surat Hotchandani, Robert Carpentier, Heidar-Ali Tajmir-Riahi

**Affiliations:** Département de Chimie-Biologie, Université du Québec à Trois-Rivières, Trois-Rivières, Québec, Canada; University of Hyderabad, India

## Abstract

Lead is a potent environmental toxin that has accumulated above its natural level as a result of human activity. Pb cation shows major affinity towards protein complexation and it has been used as modulator of protein-membrane interactions. We located the binding sites of Pb(II) with human serum (HSA) and bovine serum albumins (BSA) at physiological conditions, using constant protein concentration and various Pb contents. FTIR, UV-visible, CD, fluorescence and X-ray photoelectron spectroscopic (XPS) methods were used to analyse Pb binding sites, the binding constant and the effect of metal ion complexation on HSA and BSA stability and conformations. Structural analysis showed that Pb binds strongly to HSA and BSA *via* hydrophilic contacts with overall binding constants of K_Pb-HSA_ = 8.2 (±0.8)×10^4^ M^−1^ and K_Pb-BSA_ = 7.5 (±0.7)×10^4^ M^−1^. The number of bound Pb cation per protein is 0.7 per HSA and BSA complexes. XPS located the binding sites of Pb cation with protein N and O atoms. Pb complexation alters protein conformation by a major reduction of α-helix from 57% (free HSA) to 48% (metal-complex) and 63% (free BSA) to 52% (metal-complex) inducing a partial protein destabilization.

## Introduction

Pb is a potent environmental toxin that has accumulated 1000-fold above its natural level as a result of human activity [1]. Even though the deadly effects of lead on human health have been known for many years, the molecular mechanism of Pb toxicity is poorly understood. It is known that Pb cation mimics the effects of Ca and Zn at specific molecular targets [2], [3]. Lead toxicity has multifunctional effects on both *in vivo* and *in vitro* photosynthetic CO_2_ fixation and long term exposure results in reduced leaf growth, decreased level of photosynthetic pigments, altered chloroplast structure and decreased enzymatic activity of CO_2_ assimilation [4], [5]. Furthermore, photosynthesis is one of the most Pb-sensitive process in plants [5]. Therefore to better understand the interaction of Pb with proteins it was of interest to study the complexation of Pb with well known model proteins such as human and bovine serum albumins and determine Pb binding sites and the effect of metal ion interaction on protein structures in aqueous solution.

**Figure 1:**
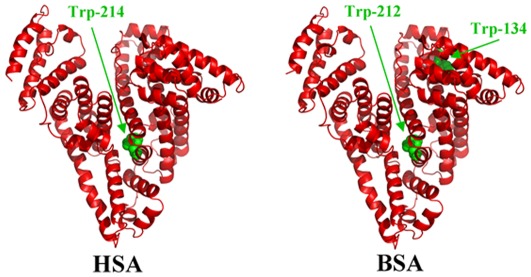
Three-dimensional structures of HSA and BSA with tryptophan residues in green color.

Serum albumins are the major soluble protein constituents of the circulatory system and have many physiological functions [6]. The most important property of this group of proteins is that they serve as transporters for a variety of organic and inorganic compounds including metal ions. BSA (Fig. 1) has been one of the most extensively studied of this group of proteins, particularly because of its structural homology with human serum albumin (HSA). The BSA molecule is made up of three homologous domains (I, II, III) which are divided into nine loops (L1–L9) by 17 disulfide bonds. The loops in each domain are made up of a sequence of large-small-large loops forming a triplet. Each domain in turn is the product of two subdomains (IA, IB, etc.). X-crystallographic data [7] show that the albumin structure is predominantly α-helical with the remaining polypeptide occurring in turns and extended or flexible regions between subdomains with no β-sheets. BSA (Fig. 1) has two tryptophan residues that possess intrinsic fluorescence [8]. Trp-134 in the first domain and Trp-212 in the second domain.Trp-212 is located within a hydrophobic binding pocket of the protein and Trp-134 is located on the surface of the molecule. HSA (Fig. 1) is a globular protein composed of three structurally similar domains (I, II and III), each containing two subdomains (A and B) and stabilized by 17 disulphide bridges [9]–[10]. Aromatic and heterocyclic ligands were found to bind within two hydrophobic pockets in subdomains IIA and IIIA, namely site I and site II [8]–[10]. Seven binding sites for fatty acids are localized in subdomains IB, IIIA, IIIB and on the subdomain interfaces [8]. While there are marked similarities between BSA and HSA in their compositions (Fig. **1**), HSA has only one tryptophan residue Trp-214, while BSA contains two tryptophan Trp-212 and Trp-134 as fluorophores.

Fluorescence quenching is considered as a useful method for measuring binding affinities. Fluorescence quenching is the decrease of the quantum yield of fluorescence from a fluorophore induced by a variety of molecular interactions with quencher molecule [11]. Therefore, using the quenching of the intrinsic tryptophan fluorescence of BSA (Trp-212 and Trp-134) and HSA (Trp-214) as a tool allows us to study the interaction of lead cation with serum proteins in an attempt to characterize the nature of Pb-protein complexation.

In this report, we present spectroscopic analysis and XPS study of the interaction of Pb(II) with HSA and BSA in aqueous solution at physiological conditions, using constant protein concentration and various metal ion contents. Structural information regarding Pb binding site and the effect of metal-protein complexation on the stability and conformation of HSA and BSA is also reported here.

## Materials and Methods

### Materials

HSA and BSA fraction V and PbCl_2_ were purchased from Sigma Chemical Company (St-Louise, MO) and used as supplied. Other chemicals were of reagent grades and used as supplied.

### Preparation of stock solutions

Protein (BSA or HSA) was dissolved in aqueous solution (40 mg/ml or 0.5 mM) containing 10 mM Tris-HCl buffers (pH 7.2). The protein concentration was determined spectrophotometrically using the extinction coefficient of 36 500 M^−1^ cm^−1^ at 280 nm [12]. A PbCl_2_ solution of 1 mM was prepared in 10 mM Tris-HCl and diluted to various concentrations in Tris-HCl (pH 7.2).

### FTIR spectroscopic measurements

Infrared spectra were recorded on a FTIR spectrometer (Impact 420 model), equipped with deuterated triglycine sulphate (DTGS) detector and KBr beam splitter, using AgBr windows. Solution of PbCl_2_ was added dropwise to the protein solution with constant stirring to ensure the formation of homogeneous solution and to reach the target Pb concentrations of 0.125, 0.25 and 0.5 mM with a final protein concentration of 0.25 mM. Spectra were collected after 2 h incubation of HSA or BSA with Pb solution at room temperature, using hydrated films. Interferograms were accumulated over the spectral range 4000–600 cm^−1^ with a nominal resolution of 2 cm^−1^ and 100 scans. The difference spectra [(protein solution+Pb solution)−(protein solution)] were generated using water combination mode around 2300 cm^−1^, as standard [13]. When producing difference spectra, this band was adjusted to the baseline level, in order to normalize difference spectra.

### Analysis of protein conformation

Analysis of the secondary structure of HSA and BSA and their PbCl_2_ complexes was carried out on the basis of the procedure previously reported [14]. The protein secondary structure is determined from the shape of the amide I band, located around 1650–1660 cm^−1^. The FTIR spectra were smoothed and their baselines were corrected automatically using Grams AI software. Thus the root-mean square (rms) noise of every spectrum was calculated. By means of the second derivative in the spectral region 1700–1600 cm^−1^ six major peaks for HSA, BSA and complexes were resolved. The above spectral region was deconvoluted by the curve-fitting method with the Levenberg-Marquadt algorithm and the peaks corresponding to *α*-helix (1660–1654 cm^−1^), *β*-sheet (1637–1614 cm^−1^), turn (1678–1670 cm^−1^), random coil (1648–1638 cm^−1^) and *β*-antiparallel (1691–1680 cm^−1^) were adjusted and the area was measured with the Gaussian function. The areas of all the component bands assigned to a given conformation were then summed up and divided by the total area [15], [16]. The curve-fitting analysis was performed using the GRAMS/AI Version 7.01 software of the Galactic Industries Corporation.

### Circular dichroism

CD Spectra of HSA, BSA and their Pb complexes were recorded with a Jasco J-720 spectropolarimeter. For measurements in the far-UV region (178–260 nm), a quartz cell with a path length of 0.01 cm was used in nitrogen atmosphere. Protein concentration was kept constant (12.5 µM), while varying PbCl_2_ concentrations (0.125, 0.25 and 0.5 mM). An accumulation of three scans with a scan speed of 50 nm per minute was performed and data were collected for each nm from 260 to 180 nm. Sample temperature was maintained at 25°C using a Neslab RTE-111 circulating water bath connected to the water-jacketed quartz cuvettes. Spectra were corrected for buffer signal and conversion to the Mol CD (Δε) was performed with the Jasco Standard Analysis software. The protein secondary structure was calculated using CDSSTR, which calculates the different assignments of secondary structures by comparison with CD spectra, measured from different proteins for which high quality X-ray diffraction data are available [17], [18]. The program CDSSTR is provided in CDPro software package which is available at the website: http://lamar.colostate.edu/~sreeram/CDPro.

### Fluorescence spectroscopy

Fluorimetric experiments were carried out on a Varian Cary Eclipse. Solutions containing PbCl_2_ 1 to 100 µM in Tris-HCl (pH = 7.4) were prepared at room temperature (24±1°C). Solutions of HSA and BSA containing 7.5 µM in 10 mM Tris-HCl (pH = 7.2) were also prepared at 24±1°C. The fluorescence spectra were recorded at λ_exc_ = 280 nm and λ_em_ from 287 to 500 nm. The intensity at 347 nm (tryptophan) was used to calculate the binding constant (*K*) according to previous literature reports [19]–[24].

On the assumption that there are (*n*) substantive binding sites for quencher (*Q*) on protein (*B*), the quenching reaction can be shown as following.
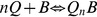
(1)The binding constant (*K_A_*), can be calculated as:
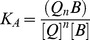
(2)Where [*Q*] and [*B*] are the quencher and protein concentration, respectively, [*Q_n_B*] is the concentration of non fluorescent fluorophore―quencher complex and [B_0_] gives total protein concentration.
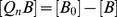
(3)

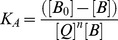
(4)The fluorescence intensity is proportional to the protein concentration as describe:
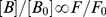
(5)Results from fluorescence measurements can be used to estimate the binding constant of Pb-protein complex. From eq 4:
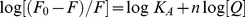
(6)The accessible fluorophore fraction (*f*) can be calculated by modified Stern-Volmer equation.
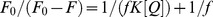
(7)Where *F*_0_ is initial fluorescence intensity and *F* is fluorescence intensities in the presence of quenching agent (or interacting molecule). *K* is the Stern-Volmer quenching constant, [Q] is the molar concentration of quencher and *f* is the fraction of accessible fluorophore to a polar quencher, which indicates the fractional fluorescence contribution of the total emission for an interaction with a hydrophobic quencher [11]. The plot of *F_0_*/(*F_0_*−F) vs 1/[Q] yields *f* ^−1^ as the intercept on *y* axis and (*f K*)^−1^ as the slope. Thus, the ratio of the ordinate and the slope gives K.

### X-ray photoelectron spectroscopy

XPS was performed on a Kratos Axis Ultra spectrometer (Kratos Analytical Ltd., UK), using a monochromatic Al Ka X-ray source (k = 1486.6 eV) with a power of 225 W, at a take-off angle of 90 degree relative to the sample surface. Two 250 µM of the sample was dropped on an aluminum substrate and dried in vacuum desiccator overnight to obtain a thin film. The dried sample was then transferred to the XPS sample holder. The measurements were made under a high vacuum of 10^−9^ torr, at room temperature. The surface of the sample was 20 mm^2^, and the investigated area was typically 192 mm^2^. Survey spectra for each sample over a binding energy range of 0–1300 eV were an average of three scans (at three different points) acquired at pass energy of 160 eV and resolution of 1 eV/step (lens in hybrid mode, which assures maximum sensitivity). High-resolution spectra of C 1 s, N 1 s and O 1 s were an average of five scans acquired at a pass energy of 40 eV and resolution of 0.1 eV/step, for quantitative measurements of binding energy and atomic concentration. Because of the potential degradation of the surface during X-ray exposure, the spectra were collected in the same order (survey, C 1 s, O 1 s, N 1 s) such that the amount exposure to X-rays was equivalent for all analyzed samples. The CasaXPS software was used for background subtraction (Shirley-type), peak integration, fitting and quantitative chemical analysis. The C 1 s (C–C) peak at 285 eV was used to calibrate the binding energy scale. Binding energies values are given at ±0.2 eV. Gaussian peak profiles were used for spectral deconvolution of C 1 s, O 1 s and N 1 s spectra [25]–[28].

## Results and Discussion

### FTIR and CD spectra of Pb complexes with HSA and BSA

**Figure 2:**
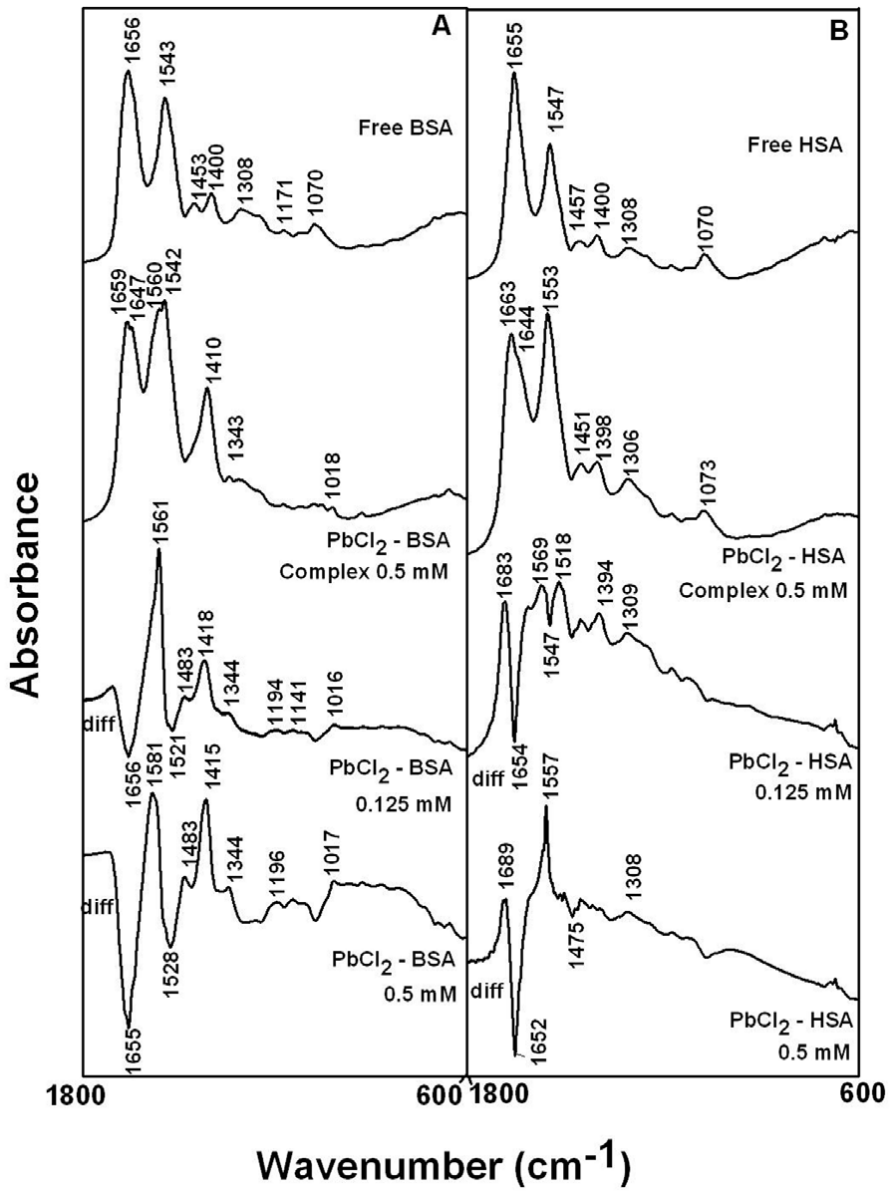
FTIR spectra in the region of 1800–600 cm^−1^ of hydrated films (pH 7.4) for free BSA (0.25 mM) and its Pb complexes (A) and for free HSA (0.25 mM) and its Pb complexes (B) with difference spectra (diff.) (bottom two curves) obtained at different Pb concentrations (indicated on the figure).

**Figure 3:**
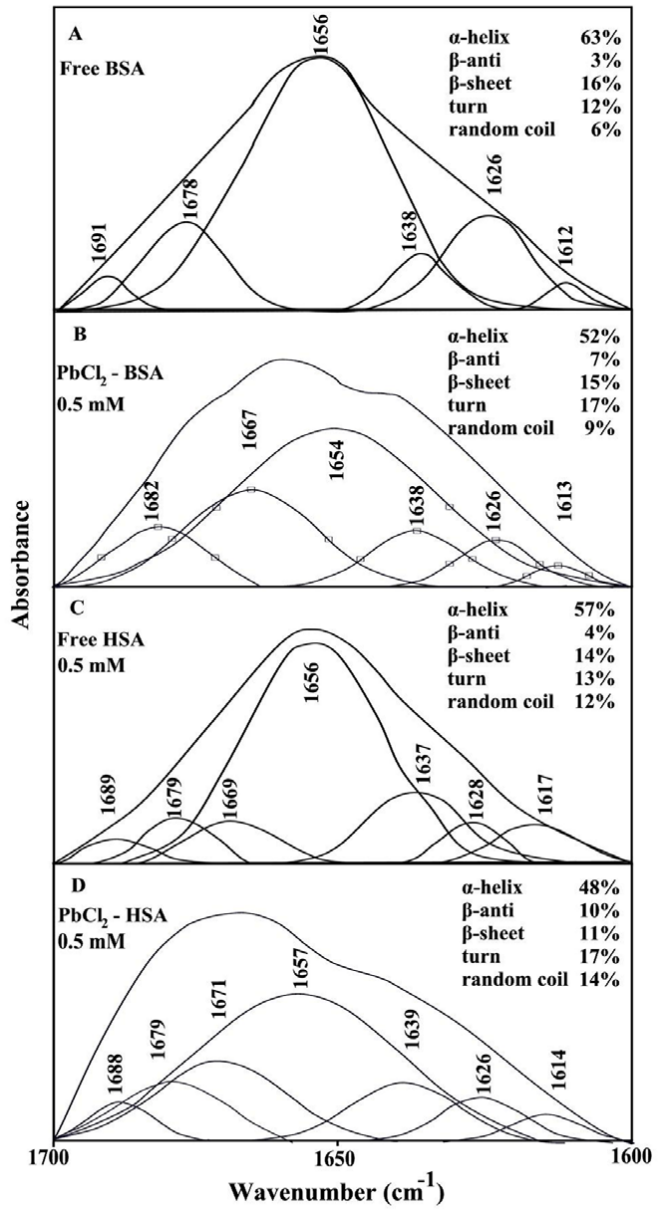
Second derivative resolution enhancement and curve-fitted amide I region (1700–1600 cm^−1^) for free BSA and HSA (0.25 mM) and their Pb complexes with 0.5 mM Pb concentration.

The Pb(II) complexation with HSA and BSA was characterized by infrared spectroscopy and its derivative methods. The spectral shifting and intensity variations of protein amide I band at 1656–1655 cm^−1^ (mainly C = O stretch) and amide II band at 1547–1543 cm^−1^ (C-N stretching coupled with N-H bending modes) [14], [15], [29] were monitored upon Pb interaction. The difference spectra [(protein solution+PbCl_2_ solution)−(protein solution)] were obtained, in order to monitor the intensity variations of these vibrations and the results are shown in Figure 2. Similarly, the infrared self-deconvolution with second derivative resolution enhancement and curve-fitting procedures [14] were used to determine the protein secondary structures in the presence of Pb cations (Figure 3 and Table 1).

**Table 1:** Secondary structure analysis (infrared spectra) for the free HSA and BSA and their Pb complexes in hydrated film at pH 7.4.

Amide I (cm^−1^) components	free BSA (%) 0.25 µM	Pb (%) 0.5 mM	free HSA (%) 0.25 µM	Pb (%) 0.5 mM
α-helix (±4) 1654–1660	63	52	57	48
β-sheet (±2) 1614–1637	16	15	14	11
random (±1) 1638–1648	6	9	12	24
turn (±2) 1970–1678	12	17	13	17
β-antiparallel (±1) 1680–1691	3	7	4	10

At low Pb(II) concentration (0.125 mM), decrease of intensity was observed for the protein amide I at 1656–1655 and amide II at 1547–1543 cm^−1^, in the difference spectra of the Pb-BSA and Pb-HSA complexes (Figs. 2A and 2B, diff. 0.125 mM). The negative features located in the difference spectra for amide I and II bands at 1656, 1561 cm^−1^ (Pb-BSA) and 1654, 1547 cm^−1^ (Pb-HSA) are due to the loss of intensity of amide I and amide II bands upon Pb interaction (Fig. 2A and 2B, diff., 0.125 mM). This reduction of the intensity for the amide I and amide II bands is due to Pb binding to protein C = O, C-N and N-H groups. Additional evidence to support the Pb interactions with C-N and N-H groups comes from the shifting of the protein amide A band at 3300 cm^−1^ (N-H stretching) in the free HSA and BSA to higher frequency at 3310–3315 cm^−1^ upon lead cation interaction (spectra not shown). As Pb concentration increased to 0.5 mM, strong negative features were observed for amide I band at 1655, 1528 cm^−1^ (Pb-BSA) and at 1652 (negative band), 1557 cm^−1^ (positive band) (Pb-HSA), upon Pb complexation (Figs. 2A and 2B, diff, 0.5 mM). In addition, spectral shifting and splitting were observed for the amide I from 1656 to 1659 cm^−1^ (Pb-BSA) and from 1655 to 1663 cm^−1^ (Pb-HSA) in the spectra of Pb-protein complexes (Fig. 2A and 2B, 0.5 mM complexes). The observed shifting and splitting of amide I band are due to Pb cation binding to protein C-O and C-N groups, while the decrease in the intensity of the amide I band in the spectra of the Pb-protein complexes suggests a major reduction of protein α-helical structure at high metal ion concentrations [30].

A quantitative analysis of the protein secondary structure for the free HSA, BSA and their Pb complexes in hydrated films has been carried out and the results are shown in Figure 3 and Table 1. The free HSA has 57% *α*-helix (1656 cm^−1^), *ß*-sheet 14% (1628 and 1617 cm^−1^), turn structure 13% (1669 cm^−1^), *ß*-antiparallel 4% (1689 cm^−1^) and random coil 12% (1637 cm^−1^) (Fig. 3C and Table 1) consistent with the spectroscopic studies of human serum albumin [15], [20]. The free BSA contains α-helix 63% (1656 cm^−1^), *ß-*sheet 16% (1612 and 1626 cm^−1^), turn 12% (1678 cm^−1^), *ß*-antiparallel 3% (1691 cm^−1^) and random coil 6% (1638 cm^−1^) (Fig. 3A and Table 1) consistent with the conformation of BSA reported [31], [32]. Upon Pb cation interaction, a major decrease of *α*-helix from 57% (free HSA) to 48% (Pb complex) with an increase in the turn structure from 13% to 17% (Pb complex) were observed (Fig. 3D and Table 1). Similarly, for BSA, a major decrease of *α*-helix from 63% (free BSA) to 52% (complex) with an increase in the turn structure from 12% to 17% (Pb complex) were observed upon Pb complexation (Fig. 3B and Table 1).

### CD spectra

The conformational changes observed from infrared results for HSA and BSA and their Pb complexes are consistent with CD spectroscopic analysis shown in Table 2. The CD results show that free BSA has a high α-helix content 60%, β-sheet 14%, turn 10% and random coil 16% (Table 2), consistent with the literature report [33]. The free HSA contains α-helix 55%, β-sheet 16%, turn 14% and random coil 15% (Table 2). Upon Pb cation complexation, major reduction of α-helix was observed from 60% in free BSA to 50% in Pb-BSA and from 55% to 45% in the Pb-HSA complexes (Table 2). The decrease in α-helix was accompanied by an increase in the β-sheet, turn and random coil structures (Table 2). The major reduction of the α-helix with an increase in the β-sheet, turn and random structures are consistent with the infrared results, indicating a partial protein destabilization (Tables 1 and 2).

**Table 2:** Secondary structure of HSA and BSA complexes (pH 7.4) with Pb cation calculated by CDSSTR Software (CD spectra).

Components conformation	free BSA (%) 12.5 µM	Pb (%) 0.5 mM	free HSA (%) 12.5 µM	Pb (%) 0.5 mM
α-helix (±3)	60	50	55	45
β-sheet (±2)	14	17	16	19
turn (±1)	10	15	14	16
random (±2)	16	18	15	20

### Fluorescence spectra and stability of Pb complexes with HSA and BSA

**Figure 4:**
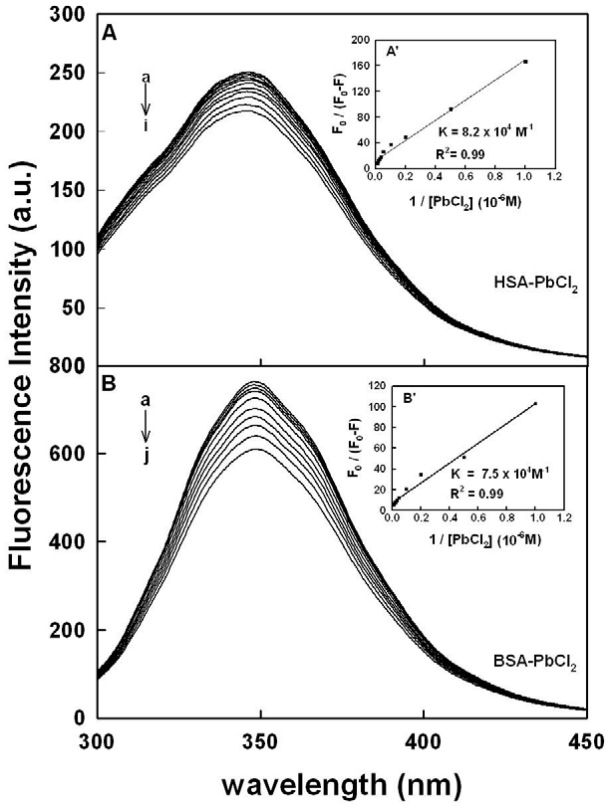
Fluorescence emission spectra of Pb-BSA systems in 10 mM Tris-HCl buffer pH 7.4 at 25°C presented for (A) Pb–BSA: (a) free BSA (7.5 µM), (b–j) with Pb cationl at 1, 2.5, 5, 7.5, 10, 15, 20, 30, 40 and 60 µM; (B) Pb– HSA: (a) free HSA (7.5 µM), (b–i) Pb at 1, 2.5, 5, 7.5, 10, 15, 20, 30, 40 and 60 µM. Inset: *F*_0_/(*F*_0_−*F*) vs 1/[Pb] for A′ (Pb-BSA) and B′ (Pb-HSA).

HSA contains a single polypeptide of 585 amino acids with only one tryptophan (Trp-214) located in subdomain II A. BSA contains two tryptophan residues Trp-134 and Trp-212 located in the first and second domains of protein hydrophobic regions. Tryptophan emission dominates both HSA and BSA fluorescence spectra in the UV region. The decrease of fluorescence intensity of HSA and BSA has been monitored at 347 nm for Pb-protein systems (Fig. 4A and 4B show representative results for each system). The plot of *F*_0_/(*F*_0_−*F*) vs 1/[Pb] (Fig. 4A′ and 4B′ show representative plots for Pb-protein complexes). Assuming that the observed changes in fluorescence come from the interaction between Pb cation and HSA or BSA, the quenching constant can be taken as the binding constant of the complex formation. The *K* values given here are averages of four-replicate and six-replicate runs for Pb-protein systems, each run involving several different Pb cation concentrations (Fig. 4A and 4B). The binding constants obtained were K_Pb-HSA_ = 8.2 (±0.8)×10^4^ M^−1^ and K_Pb-BSA_ = 7.5 (±0.7)×10^4^ M^−1^ (Fig. 4A′ and 4B′). The association constants calculated for the Pb complexes suggest strong affinity for Pb-protein binding, compared to the other ligand-protein adducts [33], [34].

**Figure 5:**
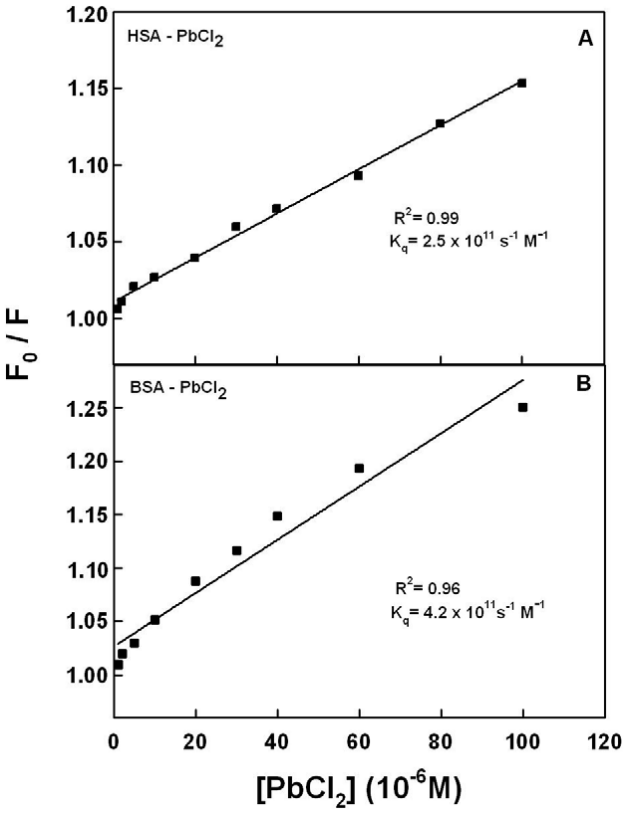
Stern-Volmer plots of fluorescence quenching constant (*K*q) for the Pb-BSA and Pb-HSA complexes at different Pb concentrations (A) Pb-BSA and (B) Pb-HSA.

In order to verify the presence of static or dynamic quenching in Pb-protein complexes we have plotted *F_0_/F* against *Q* and the results are show in Fig. 5. The plot of *F_0_/F* versus Q is linear for Pb-BSA and Pb-HSA adducts indicating that the quenching is mainly static in these Pb-protein complexes [24]. The *K*_q_ was estimated according to the Stern-Volmer equation:
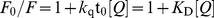
(8)where *F_0_* and *F* are the fluorescence intensities in the absence and presence of quencher, [Q] is the quencher concentration and *K*_D_ is the Stern-Volmer quenching constant (*K_q_*), which can be written as *K*_D_ = k_q_t_0_; where *k_Q_* is the bimolecular quenching rate constant and t_0_ is the lifetime of the fluorophore in the absence of quencher, 5.9 ns for BSA and 5.6 ns for HSA [10], [23], [24]. The quenching constants (*K_q_*) are 2.5×10^11^ M^−1^/s for Pb-BSA and 4.2×10^11^ M^−1^/s for Pb-HSA complexes (Fig. 5). Since these values are much greater than the maximum collisional quenching constant (2.0×10^10^ M^−1^/s), thus the static quenching is dominant in these Pb-protein complexes [35].

**Figure 6:**
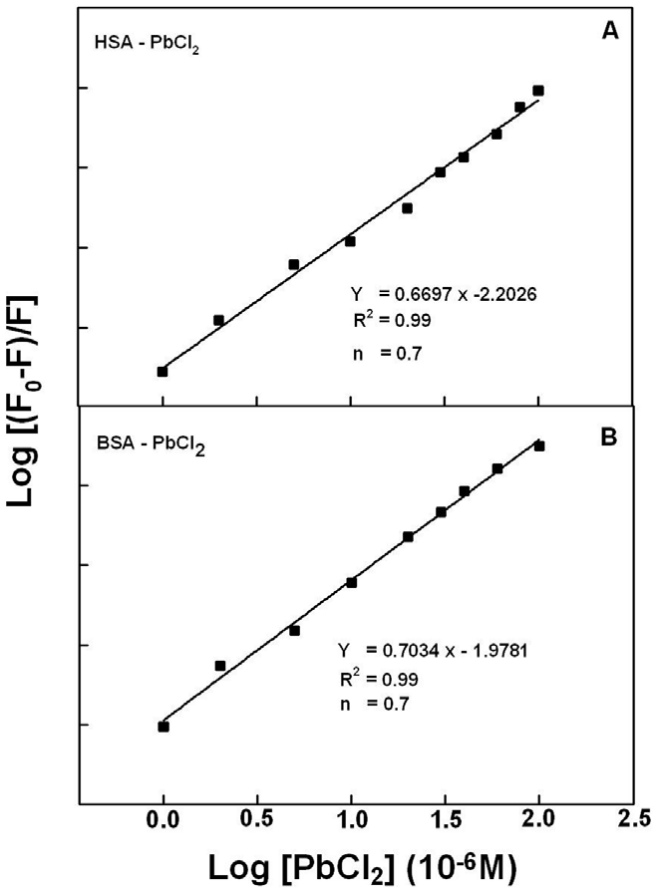
The plot of Log (*F_0_−F/F*) as a function of Log (Pb concentration).

The number of Pb cation bound per protein (*n*) is calculated from log [(*F*_0_−*F*)/*F*] = log *K*_S_+*n* log [Pb] for the static quenching [35]–[40]. The linear plot of log [(*F*_0_−*F*]/*F*] as a function of log [Pb] is shown in Figure 6A and 6B. The *n* values from the slope of the straight line are 0.7 for Pb-HSA and Pb-BSA complexes, indicating of one Pb cation bound per protein (Fig. 6A and 6B).

### XPS studies and Pb-protein binding sites

**Figure 7:**
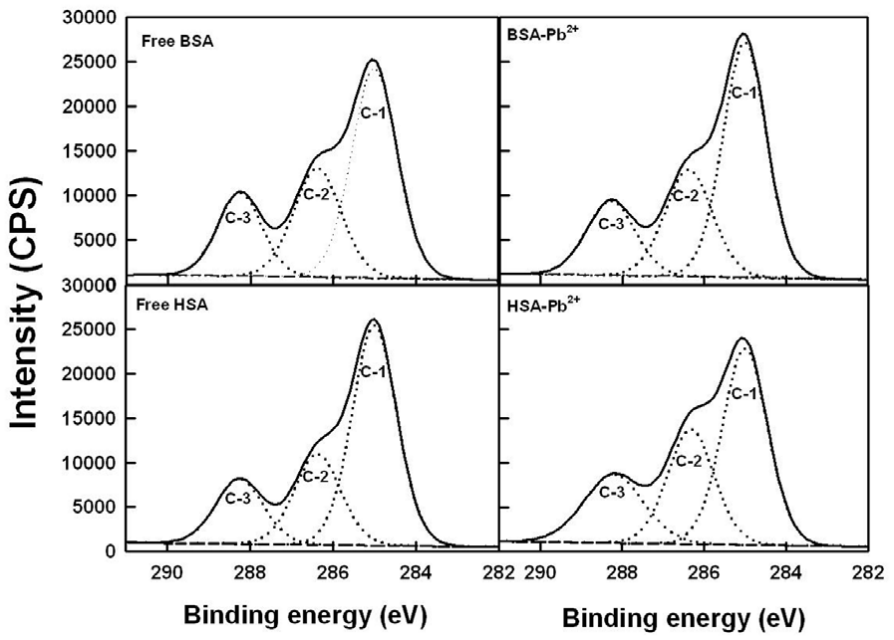
XPS spectra of C atoms for the free HSA and BSA and their Pb complexes.

**Figure 8:**
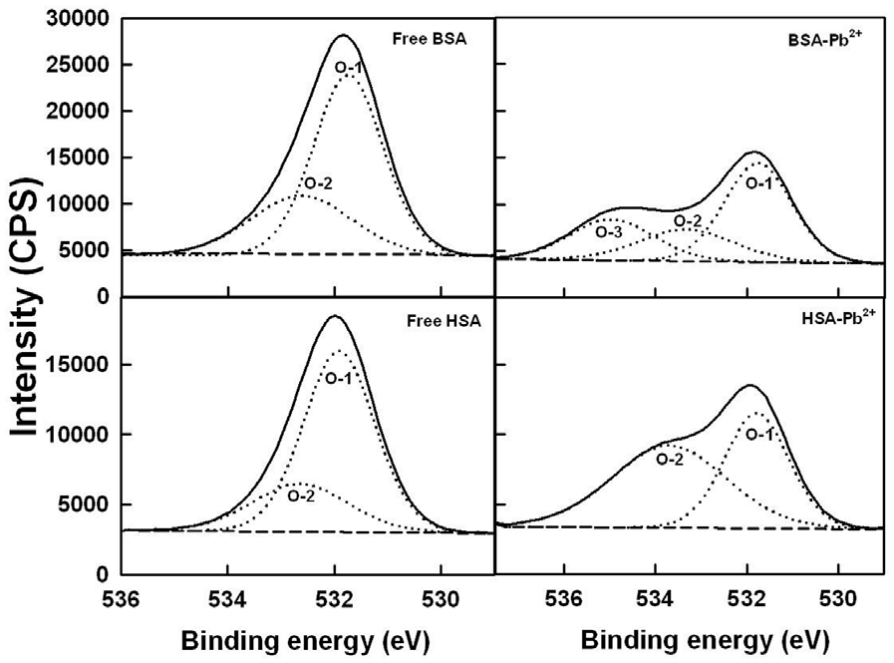
XPS spectra of O atoms for the free HSA and BSA and their Pb complexes.

**Figure 9:**
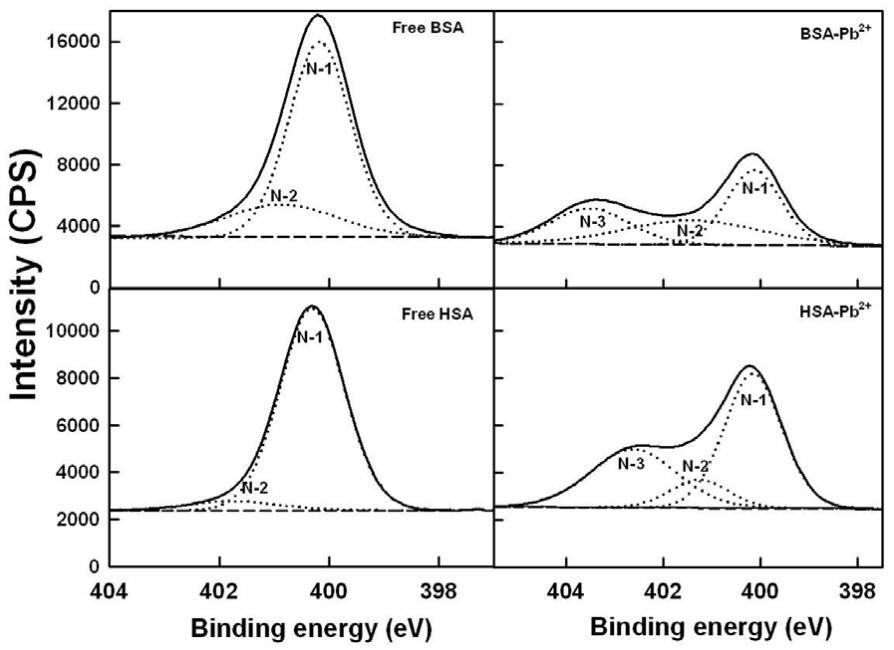
XPS spectra of N atoms for the free HSA and BSA and their Pb complexes.

To determine the Pb-protein binding sites, we have carried out X-ray photoelectron spectroscopic study. Figures 6, 7 and 8 show the XPS results. The experimental atomic composition as determined from the XPS spectral analysis and the calculated oxygen and nitrogen to carbon (ON/C) ratio for all samples are presented in Table 3. Table 3 shows the experimental atomic composition as determined from the XPS spectral analysis and the calculated oxygen and nitrogen to carbon (ON/C) ratios for all samples. All XPS spectra reveal that the C, N and O atoms are the predominant species and they occur at 285, 401 and 532 eV, respectively (Figs. 7, 8 and 9). The high resolution C-1 (C-C, C = C and C-H), C-2 (C-NH) and C-3 (C-O) of HSA and BSA are observed at about 285, 286 and 288 ev respectively (Fig. 7). The O-1 (C = O), O-2 (O-H) and O-3 (C-O) are located at about 531, 532, 534 ev, respectively (Fig. 8). Finally, N-1 (C-N), N-2 (NH_2_) and N-3 (NH_3_^+^) are positioned at 400, 401 and 402 ev, respectively (Fig. 9). These assignments are consistent with other literature reports [25]–[28], [41]. Experimental atomic compositions and O/C and N/C ratios obtained by XPS analysis for BSA, BSA-Pb, HSA and HSA-Pb are listed in Table 3. The analysis of data presented in Table 3 shows major changes in the percentages of carbon, oxygen and nitrogen atoms, while no major changes observed for sulfur atom (Table 3). It is interesting to note that the percentages of C, O and N atoms were decreased for BSA upon Pb complexation, while they increased for HSA on Pb interaction (Table 3). This could be indicative of a different binding patterns of Pb cations in BSA and HSA complexes. The major ratio changes observed for N and O are coming from direct Pb coordination with nitrogen and oxygen atoms, while the alterations of C ratios are related to the linkage of C atom to metal ion bonded N and O atoms (41). The XPS results show clearly that N and O atoms are the major metal ion binding sites in these Pb-protein complexes.

**Table 3:** Experimental atomic compositions and O/C and N/C ratios obtained by XPS analysis for BSA, Pb-BSA, and for HSA, Pb-HSA with ∼±1%.

Sample	Atomic content (%)					O/C	N/C
	C	O	N	S	Pb		
Free BSA	67. 6	18.2	13.4	0.8	0.0	0.27	0.20
Pb-BSA	71.0	16.3	11.8	0.7	0.2	0.23	0.17
Free HSA	71.7	15.9	11.7	0.7	0.0	0.22	0.16
Pb-HSA	69.2	16.9	12.9	0.8	0.2	0.24	0.19

### Conclusion

Pb cations bind strongly to HSA and BSA *via* hydrophilic interactions with overall binding constants of K_Pb-HSA_ = 8.6×10^4^ M^−1^ and K_Pb-BSA_ = 7.5×10^4^ M^−1^. The polypeptide O and N atoms are the main binding sites of Pb cations. Pb interaction alters protein secondary structure of both HSA and BSA causing a partial protein destabilization.
